# Renal Outcomes and Other Adverse Effects of Cannabinoid Supplementation

**DOI:** 10.3390/nu17010059

**Published:** 2024-12-27

**Authors:** Ewelina Młynarska, Natalia Kustosik, Maja Mejza, Zuzanna Łysoń, Dawid Delebis, Jakub Orliński, Jacek Rysz, Beata Franczyk

**Affiliations:** 1 Department of Nephrocardiology, Medical University of Lodz, 90-549 Lodz, Poland; 2 Department of Nephrology, Hypertension and Internal Medicine, Medical University of Lodz, 90-549 Lodz, Poland

**Keywords:** phytocannabinoids, endocannabinoids, synthetic cannabinoids, cannabinoid supplementation, renal pathology, acute kidney disease, kidney stone disease, diabetic nephropathy, chronic kidney disease, novel therapies, cannabinoid receptors

## Abstract

This narrative review explores the benefits and risks of cannabinoids in kidney health, particularly in individuals with pre-existing renal conditions. It discusses the roles of cannabinoid receptor ligands (phytocannabinoids, synthetic cannabinoids, and endocannabinoids) in kidney physiology. The metabolism and excretion of these substances are also highlighted, with partial elimination occurring via the kidneys. The effects of cannabinoids on kidney function are examined, emphasizing both their potential to offer nephroprotection and the risks they may pose, such as cannabinoid hyperemesis syndrome and ischemia-reperfusion injury. These complexities underscore the intricate interactions between cannabinoids and renal health. Furthermore, this review highlights the association between chronic synthetic cannabinoid use and acute kidney injury, stressing the need for further research into their mechanisms and risks. This article also highlights the growing prevalence of edible cannabis and hemp seed consumption, emphasizing their nutritional benefits, legal regulations, and challenges such as inconsistent labeling, potential health risks, and implications for kidney health. The review delves into the roles of CB1 and CB2 receptors in diabetic nephropathy, chronic kidney disease, and obesity-related kidney dysfunction, discussing the therapeutic potential of CB2 agonists and CB1 antagonists. Additionally, it examines the potential diuretic and anti-inflammatory effects of cannabinoids in preventing kidney stones, suggesting that cannabinoids could reduce crystal retention and lower the risk of stone formation. Cannabinoids’ effects on kidneys depend heavily on the characteristics of individual substances, as synthetic cannabinoids pose a major threat to the health of users. Cannabinoids offer therapeutic potential but require more research to confirm their benefits. Distinguishing between therapeutic cannabinoids and harmful synthetic variants is crucial for safe clinical application.

## 1. Introduction

*Cannabis sativa* L. (cannabis) has been cultivated since the establishment of the first agrarian societies [1]. Across different cultures, it was used to help with medical conditions, mostly being applied to relieve pain associated with diseases [2]. Cannabis, also known as marijuana, refers to the dried leaves and flowering tops of the *Cannabis sativa* or *Cannabis indica* plant [3]. Its psychoactive properties have been recognized for centuries, and as the stigma around its use diminishes, more countries are moving toward its legalization. According to a 2022 UN drug report [4], not only was marijuana the most popular drug, but its number of users increased by 23% during the years 2010–2020 [5].

Currently, marijuana is also gaining popularity in ‘alternative’ medicine due to its potential benefits in alleviating symptoms and treating certain diseases [6]. Even though marijuana is most commonly associated with smoking, other forms of intake, including direct consumption, are becoming more prevalent. For instance, among cancer patients and survivors, the most common modes of cannabis intake included ingestion of edible products through means such as topical application, smoking, and vaping [7]. Examples of cannabis products which can be ingested include tinctures, oils, and oil-filled capsules, but recreational users commonly ingest cannabis-infused foods and beverages [8]. In the USA, the foods most commonly saturated with cannabis extracts include gummies, chocolates, baked goods, fruit chews and bites, mints, and other snacks [9]. In the case of beverages, consumption due to cultural reasons is common in some Asian countries such as India (bhang) [10]. In Western countries, new alcoholic and non-alcoholic drinks containing cannabis started appearing more recently following its legalization [11]. Edibles allow users to avoid the consequences associated with smoking. Nevertheless, their use has previously been associated with a higher likelihood of overdosing and accidental ingestion [12].

Public debate rarely addresses the negative effects of cannabis on specific health conditions, such as kidney diseases, leaving a critical knowledge gap. This is particularly seen in the case of edible forms of the drug, mostly due to the inability to compare between products. This in turn derives from the lack of standardized limits and low label claim accuracy [13]. Vida et al. found that among the CBD oils available for purchase online in 2023, only half were labeled accurately [14]. The research was restricted to substances available in Hungary. Nonetheless, this clearly exhibits the problem underlying cannabis supplementation. In another study, Cohen et al. evaluated the quantity of melatonin and CBD in melatonin-enriched gummies sold in the USA [15]. All of the CBD products had doses exceeding the labels’ claims.

Delta-9-tetrahydrocannabinol (THC) is a phytocannabinoid and the constituent responsible for the psychoactive effects of cannabis. It is mostly metabolized by the liver, but about 20% of the metabolites are excreted through urine [16,17,18]. Moreover, the kidneys were shown to have some of the highest rates of synthetic cannabinoid (SC) concentrations [19]. This paper explores the mechanisms of cannabinoid metabolism and excretion via the kidneys, highlighting their potential implications for renal function.

This suggests the potential for renal complications, particularly in vulnerable populations or in individuals with pre-existing kidney conditions. This review summarizes both the positive and (particularly) the negative impacts of cannabinoids in major kidney diseases, summarizing the recent findings. Additionally, it provides a categorization of cannabinoid types and their receptors, discussing the potential clinical usefulness of cannabis-derived substances for kidney health. Furthermore, it examines the connections between cannabis and major kidney diseases, such as chronic kidney disease and acute kidney injury, presenting findings from new studies which investigated these associations. Cannabis supplementation poses significant challenges to renal health which demand further investigation.

The effects of cannabinoids on renal health are complex and influenced by factors such as their dosage, mode of administration, and individual variability. This review discusses the role of cannabinoid receptors in kidney tissues, analyzes the effects of different classes of cannabinoids on kidney health, and synthesizes findings from clinical trials and preclinical studies to evaluate the dual impacts of cannabinoid intake on kidney health. By bridging this gap in understanding, this work aims to provide an in-depth discussion on how cannabis influences kidney disorders and renal physiology, offering healthcare professionals a clearer picture of the risks and potential clinical applications of cannabinoids in renal medicine.

This research was performed with the use of the PubMed, Google Scholar and Scopus databases. The inclusion criteria included works referencing cannabinoids, kidneys or kidney disease-related health conditions which were published before October 2024 and summarized in the form of a narrative review.

## 2. Cannabinoid Molecules

### 2.1. Natural Cannabinoids

Cannabis contains many chemical compounds which exert diverse effects on cannabinoid (CB) receptors [20,21,22]. More than 100 substances which belong to the phytocannabinoids are believed to constitute the pharmacological effect of the extract from the cannabis plant. The two most investigated substances are THC and cannabidiol (CBD). There are other phytocannabinoids which are divided into groups, with the main representants being cannabichromene (CBC), cannabielsoin (CBE), cannabigerol (CBG), cannabicyclol (CBL), cannabinol (CBN), cannabinodiol (CBND), cannabitriol (CBT) and Δ8-trans-tetrahydrocannabinol (Δ8-THC) [20].

THC acts as a partial agonist of CB1, binds to CB2 and partially activates it [21].

THC is known for its psychotropic effect and may influence one’s mental state, exacerbating psychotic disorders and leading to depression. Its psychotropic effect can be explained by both its CB1 agonism and lipophilic structure, which enables THC to cross the blood–brain barrier. In the kidneys, THC is believed to stimulate prostaglandin production [22,23].

On the contrary, CBD is believed to be an inverse agonist or allosteric modulator of CB1 and CB2 [24,25]. CBD possibly mitigates some harmful effects of THC (e.g., the cataleptic effect [26]), although there is still no strong evidence that CBD alleviates THC-induced psychotic symptoms [27]. Nevertheless, the fact that there is interaction with CB1 implicates that CBD can also modulate inflammatory processes, as demonstrated in studies using in vitro and in vivo models [28,29,30,31]. The nephroprotective effect of CBD was shown in an experiment with a gentamicin-induced nephrotoxicity model. CBD administration enhanced the activity of farnesoid X receptors (FXR) [32]. An FXR is a nuclear receptor activated by bile acid, and its activation counteracts an inflammatory response. Another anti-inflammatory mechanism of CBD is crosstalk with Toll-like receptor (TLR) downstream pathways [33]. TLRs are receptors which can recognize particles of a pathogenic origin (e.g., bacterial DNA, lipopolysaccharides (LPSs), and viral RNA). It was demonstrated that hemp extracts reduce LPS-induced inflammatory cytokine expression (namely TNF- α and IL-6) in the canine cell line [34], and cannabidiol effectively reduced the LPS-induced inflammatory response in Wistar albino female rats. The reduction was reflected by lowering the level of TNF- α and caspase 3 [35]. These findings suggest that cannabinoids might also be useful in alleviating kidney pathologies in humans.

The plant has been used as a material for smoking (leaves, stems and flower buds) and producing cloth and tools. The seeds are an oil source used in food preparation as a substitute for other cooking oils. They were used for medical and nutritional purposes, as they were believed to alleviate joint pain and cognitive impairment [36]. Nowadays, they are also added to various food products such as cakes, pastries, bread, dairy and meat to make them more palatable and provide additional micronutrients. The seeds consist of fat (~30%), fiber (~25%) and protein (~20%) [37]. Indeed, studies indicate that supplementation of hemp seeds increases essential amino acids (phenylalanine, histidine and lysine) and crucial cations such as iron (Fe) and zinc (Zn) [38]. Apart from these constituents, cannabis-derived dietary products may contain a certain number of cannabinoids. Other edible parts of cannabis such as stems and leaves possess relatively high amounts of THC and CBD (even reaching 0.2% and 2%, respectively) [37]. Therefore, only its seeds are allowed for consumption [39]. The concentration of the cannabinoids varies depending on a plethora of factors, including the age, harvest time, condition of culturing (humidity, fertilizers and light amount) and storage conditions [40]. The seeds abound with neither THC nor CBD (they contain only traces of these compounds) [39]. CBG, CBN and CBC can also be detected [41]. Although THC concentrations in food are tightly regulated by law in many countries, CBD is sometimes added to hemp oil, and this combination may exert a relevant pharmacological effect.

### 2.2. Synthetic Cannabinoids

Synthetic cannabinoids (SCs) are chemicals which interact with targets in the endocannabinoid system. The illicit market abounds with SCs, which are characterized by greater agonistic effects on CB receptors than THC. Hence, these drugs demonstrate both more devastating adverse effects and more severe psychotic symptoms. Usually, solutions containing SCs consist of organic solvents (methanol, ethanol and acetone) and are sprinkled onto plant mixtures (e.g., mint, thyme leaves, flowers and barks) [42].

Cannabinoids are usually absorbed through the respiratory tract and oral mucosa when smoked, although some products are intended for oral use in tablet, capsule or powder form.

While translation of SCs’ properties into nutritional or medical practice appears to be ill-founded, the substances antagonizing CB1 are feasible options for obesity treatment and modifying dietary habits. It is known that CB1 signaling is involved in the regulation of voluntary food intake and lipogenesis [43]. Caution should be practiced since CB1 antagonists (e.g., rimonabant) may affect mental health, contributing to depression development [44]. However, in evaluating how common diagnoses of obesity are, a molecule safely suppressing CB1 signaling would be a potent tool as a supplement to functional food. Paying more attention to the role of cannabinoid receptors in homeostasis maintenance offers the prospect of manipulating these elements via food ingredients.

Recently, plenty of reports suggested an association between SC ingestion and health complications concerning not only the central nervous system but also the heart, liver and kidneys [45]. Some clinical records described acute tubular necrosis and acute interstitial nephritis upon histological examination in SC consumers [46]. Nephrotoxicity of SCs is likely due to strong CB1 agonism. Various synthetic substances exhibiting CB1 antagonism or CB2 agonism have been examined in preclinical models (e.g., rimonabant, AM251, AM1241 and AM630) [47,48,49,50]. They demonstrated the nephroprotective effect, which could be used in the future in the therapy of toxic or inflammatory renal injury.

### 2.3. Endocannabinoids

Human organisms produce their own CB ligands, and these substances belong to a network referred to as the endocannabinoid system. The best-characterized endogenously produced CB agonists are anandamide (AEA) and 2-arachidonoylglycerol (2-AG). They are created by enzymatic reactions involving lipids of the cell membrane (phosphatidylinositol 4,5-bisphosphate (PIP2), arachidonic acid (ARA) and phosphatidylethanolamine (PE)) [51]. One of the pathways for 2-AG synthesis begins with the production of phosphatidylinositol 4,5-bisphosphate (PIP2), which is cleaved by phospholipase C (PLC) [52]. This cleavage results in diacylglycerol (DAG) formation. The second step is the conversion of DAG to 2-AG via diacyloglycerol lipase. AEA is produced using PE, which merges with ARA, and this reaction is catalyzed by N-acyltransferase [53]. As a result, a product called N-arachidonoyl phosphatidylethanolamine (NAPE) is synthetized. NAPE serves as the precursor of anandamide (AEA), which is formed by NAPE-specific phospholipase D. The 2-AG is mainly hydrolyzed by monoacylglycerol lipase into ARA and glycerol, whilst AEA is degraded by the enzyme fatty acid amide hydrolase (FAAH) into ARA and ethanoloamide. These routes of production are not the only ones [52,53], but the others will not be discussed in this paper.

We note that the described pathways of the synthesis share common elements with the production of other eicosanoids. Hence, cyclooxygenase (COX) inhibitors, such as non-steroid anti-inflammatory drugs, may interfere with the synthesis–degradation rate of these endocannabinoids. On one hand, the inhibition of COX leads to a higher concentration of ARA, which is also a substrate for prostaglandin synthesis [54]. On the other hand, some COX inhibitors exhibit the ability to reduce FAAH activity, which directly elevates the level of AEA [55]. Therefore, certain effects of the endocannabinoids, which are described further, can be modulated by easily available substances.

AEA acts as a CB1 partial agonist. It also exhibits activity for CB2, although less compared with CB1. In turn, 2-AG exerts an agonistic effect on CB1 and CB2 to a greater degree than AEA [56,57]. Both bind to the transient receptor potential cation channel vanilloid 1 (TRPV1) receptor, but AEA also binds and activates some subtypes of the peroxisome proliferator-activated receptor (PPAR) family [58]. There are also less-investigated endocannabinoids which interact with CB receptors, and this group includes N-arachidonoyl dopamine (NADA) [59] and 2-arachidonyl glyceryl ether [51,60]. Some others are presented in Table 1. More studies are required to assess their roles in healthy kidneys.

As noted, CBs are also expressed in the kidneys. Renal CB1 expression occurs in podocytes, mesangium, intercalated cells of the collecting duct, proximal convoluted and distal tubules, and afferent and efferent arterioles [89,90]. As mentioned earlier, CB2 expression in the kidneys remains controversial, but some studies indicate that podocytes [91] and proximal tubule cells [92] exhibit CB2 expression.

## 3. Cannabinoid Interaction with Renal Physiology

### 3.1. Cannabinoid Receptors

Cannabinoid receptors, both CB1 and CB2, are widely expressed in the human body. They are G protein-coupled receptors (GPCRs). GPCRs are transmembrane receptors usually containing seven lipophilic domains and a heterotrimeric complex of three units: G_α_, G_β_, and G_γ_. The G_α_ unit may occur in various forms (Gs, Gi, or Gq) and influences intracellular cAMP levels (Gs and Gi) or acts through IP3 and DAG elevation (Gq) as a second mediator in cellular downstream pathways. Both CB1 and CB2 bind Gi, and hence their activation leads to a decrease in the intracellular cAMP level. However, CB1 may sometimes bind Gs or Gq proteins [93]. The Gβ-Gγ complex of CB plays an important role in ion channel blockage and opening and the mitogen-activated protein kinase (MAPK) cascade. CB1 activation is reported to negatively regulate some types of calcium channels (N type, P/Q type, and R type) and positively regulate inwardly rectifying potassium channels [94,95]. CB regulates the activity of MAPK, which includes extracellular signal-regulated kinases (ERKs), c-Jun N-terminal kinases (JNKs), and p38 mitogen-activated protein kinases (p38s) [96]. MAPK is crucial for the survival cascade of the cell and its metabolism.

CB1 is expressed predominantly in the central nervous system (CNS), although evidence of CB1 transcription can be detected in many other organs (e.g., the kidneys, adrenal gland, heart, lungs, reproductive organs, liver, and gastrointestinal tract) [90,94,97,98] and certain immune cells [94,99]. In turn, CB2 was thought to be expressed mainly in immune cells (macrophages, NK cells, and lymphocytes) [94]. To date, CB2 has been discovered in many other sites such as the pancreas, lungs, and reproductive organs [58,91,92,100,101]. The presence of CB2 in the kidneys is debatable [102], but many authors reported its detection [91,92]. It is claimed that CB1 exerts a rather pro-inflammatory and pro-fibrogenic effect (which does not apply to CNS, however), whereas CB2 exerts an anti-inflammatory effect [89].

### 3.2. The Endocannabinoid System

**Figure 1:**
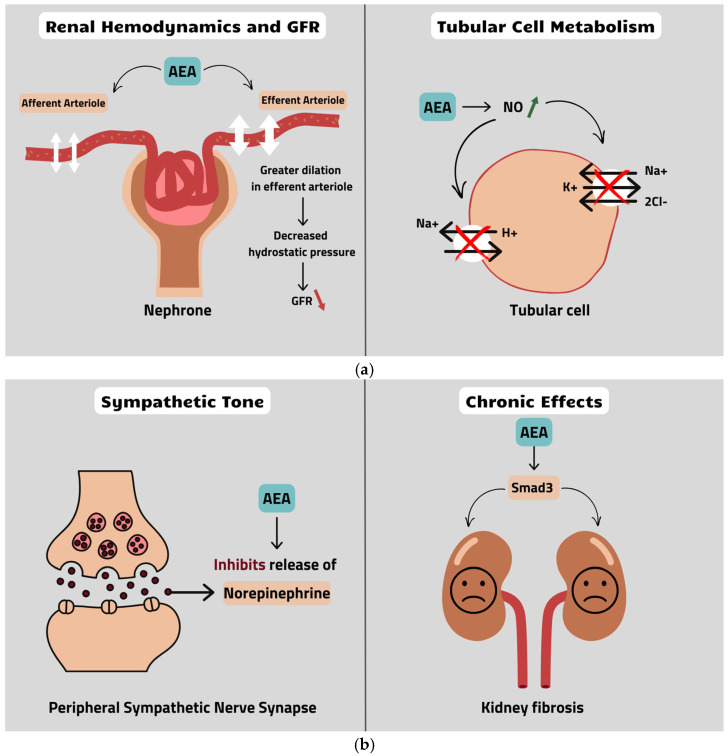
Schematic summary of effects of AEA on kidney function and structure. (**a**) Renal hemodynamics and GFR and tubular cell metabolism. (**b**) Sympathetic tone and chronic effects. AEA = anandamide; GFR = glomerular filtration rate; NO = nitric oxide; AEA = anandamide. Red arrows signify decrease, green arrows increase in certain substances and red cross a blockage.

The endocannabinoid system is responsible for regulating renal hemodynamics. We note that the relative levels of endocannabinoid (namely the AEA/2-AG ratio) may differ in some regions in the kidneys. For instance, the AEA concentration is higher in the renal medulla than in the cortex, whereas the concentration of 2-AG in the medulla and the cortex is comparable and equal to the cortical concentration of AEA [103,104,105]. AEA plays a significant role in kidney functions, influencing the glomerular filtration rate (GFR). This effect is believed to relate to CB1 agonism of AEA, which at the temporary local administration causes vasodilation in the kidneys [104,105]. Mesangial and endothelial cells are involved in the response due to their ability to synthesize and metabolize AEA [104,105]. The coexistence of a decrease in GFR and vasodilation of arterioles may seem paradoxical. This can be explained by the fact that AEA dilates efferent arterioles to a greater degree than afferent arterioles. Overall, this results in decreased hydrostatic pressure in the glomerular capillaries [106], which affects the flow of blood in the kidneys and reduces the GFR (Figure 1a).

There are a few possible mechanisms which lead to this vasodilatory effect. Firstly, AEA increases nitric oxide production, which is a potent vasodilating agent [107]. This effect is attributed to TRPV1 rather than CB1 activation [108]. Secondly, AEA exhibits an inhibitory effect on norepinephrine release in the peripheral sympathetic nerves [109] (Figure 1b). Since the sympathetic nervous system is responsible for enhancing GFR via adrenergic stimulation, another mechanism of action is the TPRV1-dependent release of calcitonin gene-related peptide (CGRP) [110], which is also released in the kidneys and acts as a vasorelaxant [111].

AEA’s influence on diuresis remains unclear. A decrease in GFR reduces urine production. Nonetheless, some studies imply that AEA may enhance diuresis. AEA modifies tubular cell metabolism by reducing oxygen consumption, which was observed in a rat experiment. Decreased oxygen consumption is probably linked to a nitric oxide increase due to AEA. Additionally, nitric oxide leads to Na^+^/H^+^ transporter and Na^+^/K^+^/2Cl^−^ co-transporter inhibition [112], as shown in Figure 1a. This effect is a key point in the activity of loop diuretics, with highly potent agents increasing urine production. Another study conducted by Ritter et al. also indicated that sodium reabsorption is controlled by AEA [103], but not all studies support this [105,113]. The role of 2-AG in healthy kidneys is still not fully known, although some studies indicate its importance in renal pathologies.

Endocannabinoid disbalance may lead to an inflammatory response and damage to organs.

It is worth mentioning that chronic AEA administration may induce fibrosis, and this phenomenon was shown in an experiment conducted by Golosova et al. [113], who observed increased activity in the Smad3 signaling pathway (Figure 1b). Another important observation was that chronic AEA intravenous administration exacerbated hypertension.

It is known that elevated 2-AG levels in the kidneys accompany renal injury [114,115]. In an experiment conducted by Zhou et al. [116], 2-AG elevation accelerated fibrosis and lipotoxicity. This molecule’s precise role has yet to be elucidated, although it seems that the duration of exposure to 2-AG plays a crucial role here.

### 3.3. Excretion and Metabolism

As previously mentioned, about 20% of THC metabolites are excreted in urine (20–35%) [117]. This is due to the high lipophilicity of THC, which leads to significant renal tubular reabsorption and limited excretion of the unchanged drug [117]. Similar pharmacokinetic properties were observed for other cannabinoids, with studies suggesting that they are primarily excreted via feces [118].

It is impossible to discuss the excretion of cannabinoids without addressing their metabolism. Cannabinoids are mainly metabolized in the liver. THC is metabolized by microsomal hydroxylation and oxidation [119]. The process is catalyzed by enzymes of the cytochrome P450 (CYP) complex. The specific enzymes responsible for the hepatic metabolism of THC are CYP2C and CYP3A. These enzymes turn THC into 11-Hydroxy-Δ9-tetrahydrocannabinol (11-OH-THC), excreted mainly through feces, and 11-Nor-9-carboxy-Δ9-tetrahydrocannabinol (11-COOH-THC), excreted mainly via urine [118].

The metabolism of CBD is also associated with the CYP complex. CBD is metabolized to the active metabolite 7-Hydroxy-CBD (7-OH-CBD) by the enzymes CYP2C19 and CYP3A4. Studies suggest that CYP2C9 may also play a significant role in metabolizing CBD to the active 7-OH-CBD [120].

The involvement of the kidneys in the excretion and metabolism of CBD and THC may be significant to optimizing the planning of therapies for kidney diseases using these substances.

### 3.4. Effect on Kidney Function

As was mentioned, THC and CBD interact with two distinct receptors in the kidneys: CB1 and CB2 [121]. Studies on animals indicate that blocking CB1 may decrease albuminuria and renal fibrosis. CB2 activation may potentially be protective, as described earlier [122].

Vomiting is sometimes listed as one of the negative effects of synthetic cannabinoids [123]. While they are generally used to prevent vomiting, there are reported cases where they have the opposite effect [124].

Chronic use of Indian hemp was connected to pre-renal kidney injury associated with cannabinoid hyperemesis syndrome. One of the symptoms of this syndrome is the previously mentioned vomiting [124]. It can be assumed that intractable vomiting may affect renal blood flow [117].

CBD may be significant in the ischemia-reperfusion mechanism [124]. It has been demonstrated by Fouad et al. that CBD administered just before reperfusion has a protective effect on renal tissue. The research was conducted on rats. The renal parameters and kidney histopathology were examined in rats treated with CBD and in a control group [125]. There was a reduction in inflammation and oxidative damage. The rats treated with CBD by Soares et al. showed lower myeloperoxidase activity and decreased levels of TNF-alpha and IL-1beta cytokines. Additionally, malondialdehyde, a marker of lipid peroxidation and protein carbonylation, was also lower compared with animals subjected to ischemia and reperfusion without CBD administration [126]. This suggests that CBD may play an important role in protecting the kidneys from the adverse effects of reperfusion. Its anti-inflammatory and antioxidative actions are significant.

THC has been linked to a dose-dependent temporary increase in heart rate and an elevation in blood pressure [127]. Murray et al., in a case crossover study, noted that compared with non-users, marijuana users were less likely to have a history of hypertension (30% versus 44%; *p* = 0.002) [128]. Nevertheless, a definitive association with hypertension has not been established. Orthostatic hypotension was reported with self-reported acute cannabis [129]. This may limit the scope of cannabinoid usage in kidney diseases. Nonetheless, tolerance develops after 1–2 days of repeated exposure, resulting in reversal of the previously described negative effects [117]. Some studies suggest that CBD may have a blood pressure-lowering effect [130,131,132]. The available studies do not provide clear information regarding the impact of cannabinoids on kidney perfusion or overall function over longer observation periods. Thus, further research is needed.

## 4. Adverse Renal Effects Associated with Cannabinoid Use

### 4.1. Diabetic Nephropathy

Diabetes mellitus (DM) is one of the most common chronic diseases, globally rising to a rate of 10.5% among people 20–79 years old [133]. Its progression can lead to multiple and severe complications, one of which is diabetic nephropathy (DN). This can be caused by both types of DM and is present in as many as 40% of people suffering from this disease [134]. Multiple mediators and complex pathways have been proven to have roles in its development [135]. Until recently, little attention has been paid to cannabinoids’ involvement in both the origin as well as the management of DN.

**Figure 2:**
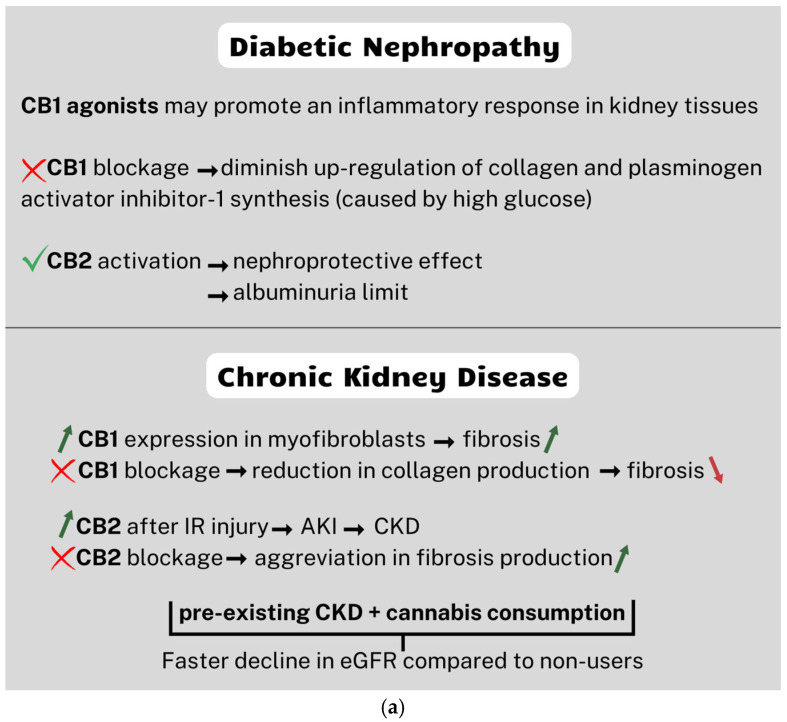

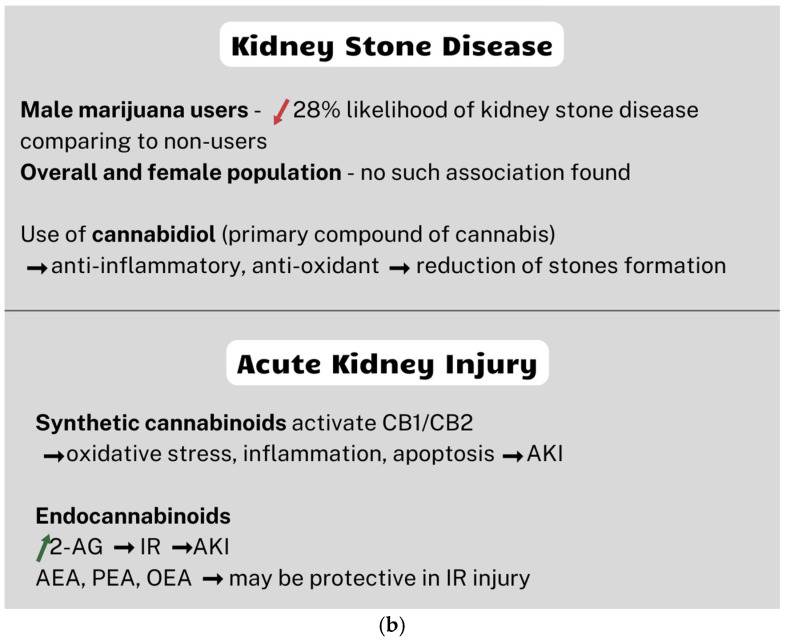
Summary of key mechanisms of cannabinoids in renal pathologies: (**a**) diabetic nephropathy and chronic kidney disease and (**b**) kidney stone disease and acute kidney injury. 2-AG = 2-arachidonoylglycerol; AEA = anandamide; AKI = acute kidney injury; CB1 = cannabinoid receptor 1; CB2 = cannabinoid receptor 2; CKD = chronic kidney disease; eGFR = estimated glomerular filtration rate; IR = ischemia and reperfusion; OEA = N-oleoylethanolamine; PAE = N-palmitoylethanolamine; CBD = cannabidiol. Red arrows signify decrease, green arrows increase in certain substances and red cross a blockage.

Previously, researchers have examined elevated CB1 expression in the podocytes of patients with DN. As noted, CB1 agonists may promote an inflammatory response in tissues, and this statement may be supported by studies conducted by Barutta et al., who with the use of the CB1 antagonist AM251 established that such a blockade prevents the loss of nephrin, podocin, and zonula occludens-1 (Figure 2a) [48].

This in turn may explain why the use of AM251 also improved albuminuria, which was significantly increased in diabetic mice, and reduced apoptosis. Further studies proved that CB1 blockading can protect db/db mice by diminishing the upregulation of collagen and plasminogen activator inhibitor-1 synthesis, which are caused by high glucose levels [136]. On the contrary, CB2 was shown to be downregulated in kidney biopsies of patients with advanced DM [91].

CB2 activation demonstrated a nephroprotective effect in renal disorders in mouse models, decreasing proinflammatory cytokines and reducing apoptosis [137,138]. Moreover, some studies showed improvement in albuminuria in renal dysfunction.

Therapy with the CB2 agonist was established to limit albuminuria in obese animal models [139] and in a mouse model of diabetic nephropathy [91], which opened new perspectives to the treatment of patients with diabetes. Most recent studies showed the CB2 antagonist’s potential and came to the conclusion that it may ameliorate DN in rats [140].

Multiple genetic analyses have been published as part of a search for explanations for these phenomena [141,142,143,144]. One of them [143] established a genome-wide association study, which identified a variant in the gene encoding of CB2 related to DN in the United Arab Emirates and Japanese cohort. Nonetheless, a similar association failed to be demonstrated in European populations. Conversely, a study performed on a Polish cohort found that rs10493353 (G1359A) polymorphism in the CB1 gene is associated with diabetic nephropathy and diabetic retinopathy in patients with type 2 DM (T2DM) [142]. A similar study was conducted on a northern Chinese population [144], confirming that an allele of the CB1 gene rs10493353 may potentially be protective of DN in type 2 DM patients. Nevertheless, further research and clinical trials need to be performed to draw any conclusions from these data.

### 4.2. Chronic Kidney Disease

Chronic kidney disease is an irreversible, progressive clinical syndrome [145] characterized by extracellular matrix substituting a functional renal tissue [146]. It can result from various chronic pathologies. An urgent need for new medications to help with its progression has led researchers to target the endocannabinoid system.

Analogous to DN, CB1 was shown to be overexpressed in patients with other non-metabolic CKDs [147]. Most importantly, increased expression was shown in myofibroblasts, the cells most crucial to the process of fibrosis. The latter study additionally established that the blockade of CB1 led to a reduction in collagen expression in vitro. Similar results were obtained by Dao et al. in patients with chronic allograft dysfunction, a leading cause of transplant failure [148], suggesting yet another therapeutic potential for CB1 antagonists. The relation between CB2 and renal fibrogenesis seems to be more complex. Its role in CKD has been considered controversial for some time. The latest studies have shown that CB2 is increased time-dependently after a 3 day period of ischemia-reperfusion injury [50]. This fact is crucial, considering that at this point, acute injury can progress into CKD. In contrast to this, Lecru et al. reported that CB2 blockading using synthetic antagonists has aggravated fibrosis progression [147].

The study conducted a post hoc analysis using data from the ASSESS-AKI study to assess the impact of cannabis consumption on kidney health outcomes over an average follow-up period of 4.5 years. Among the participants without chronic kidney disease, cannabis use was not associated with significant declines in the estimated glomerular filtration rate (eGFR) or an increased risk of CKD. However, in individuals with pre-existing CKD, cannabis consumption was linked to a faster annual decline in the eGFR compared with non-users, although it did not independently increase the risk of progressive CKD (Figure 2a). Further research is necessary to understand cannabis consumption’s impact, particularly within the context of existing kidney disease and potential long-term consequences [149].

Obesity is a growing public health problem which is correlated with other diseases. The disease has been described as a proposed target for CKD reduction in the global population. It has also been established that obese patients have a higher likelihood of CKD progression [150]. Moreover, studies have reported alterations in the kidney endocannabinoid system in obese mice models [151]. Obesity has previously been linked to lipid accumulation in animal models [152]. Despite it not being a well-described phenomenon in humans, it is an important factor to note when referencing the subject. The accumulation of lipids may lead to renal lipotoxicity and, in turn, fibrosis. Udi et al. established an association between obesity-related renal toxicity and the activation of CB1 [151]. Even though CB1 depletion in the renal proximal tubule was not shown to lead to any beneficial effects regarding obesity itself, it was associated with the lessening of lipid accumulation. Nevertheless, there is a need for further studies to assess the exact mechanisms of the described phenomena.

### 4.3. Kidney Stone Disease

Kidney stone disease, also called nephrolithiasis or urolithiasis, is a condition characterized by the formation of solid crystal aggregates in the urinary tract. The risk factors of this condition are genetic predisposition, metabolic factors, and the environment [153]. Importantly, kidney stones are not solely isolated issues; they are often indicators of broader systemic health problems [153].

A cross-sectional study based on data obtained from the National Health and Nutrition Examination Survey was conducted to determine if marijuana use is related to kidney stones. Marijuana use was assessed through questionnaires, categorizing participants as frequent users or non-users based on frequency and duration. Kidney stone history was determined via self-reporting. Covariates, including demographics, lifestyle, and medical factors, were considered to adjust for potential confounders in the analysis.

The NHANES survey found that the men who used marijuana had a 28% lower likelihood of reporting kidney stones compared with non-users (Figure 2b). The diuretic effects of cannabinoids shorten the time crystals stay in the kidneys, which decreases the risk of developing kidney stones [154]. However, this association was not found in the overall or female populations.

This study examined the relationship between marijuana use and kidney stones using a nationally representative NHANES dataset. It assessed both the history and frequency of use. Comprehensive adjustment for confounders and robust sensitivity analyses enhanced its reliability. A large sample size ensured generalizability, and gender-specific analyses revealed population differences.

It should be mentioned that this was a cross-sectional study, and there was a possibility of selection bias. In addition, there was no association between more frequent (≥7 times/week) marijuana use and kidney stones. These results do not provide strong evidence but can be taken into consideration.

Previous research has demonstrated that cannabinoids can increase urine output in mice without affecting the excretion of Cl^−^ and K^+^ [155]. The urine output was measured over 6 h with no access to food or water, using weigh boats to collect and weigh voided urine in 2 h intervals to minimize evaporation. Drug and vehicle doses were randomized and administered subcutaneously, with combination treatments delivered in split volumes to reduce time-dependent biases.

This study demonstrates that cannabinoid agonists induce diuresis in mice, with a biphasic effect where lower doses increase urine output via the central CB1 receptors and higher doses reduce output, possibly involving peripheral CB1 receptors. The diuretic effect at lower doses was weakly natriuretic without altering chloride or potassium excretion. These findings suggest a complex role of cannabinoid receptors in regulating water balance.

The effect shown above may shorten the duration of crystal retention in the kidneys, which can potentially lower the risk of kidney stone development, as was said in an update on the current concepts of kidney stone disease in Adv Urol. 2018 [156]. In Adv Nutr. 2023, it was also underlined that low urine output is one of the causes of nephrolithiasis [157].

What is more, cannabidiol, a primary component of cannabis, has anti-inflammatory and antioxidant abilities [136]. Inflammation and oxidative stress have crucial roles in the formation of kidney stones [158,159]. Therefore, it is possible that cannabidiol mitigates inflammatory responses and reduces oxidative stress, potentially contributing to a lower risk of kidney stone development (Figure 2b) [154].

Given the potential diuretic and anti-inflammatory effects of cannabinoids, additional research is necessary to investigate their role in kidney stone formation and prevention.

### 4.4. Acute Kidney Injury Induced by Synthetic Cannabinoids

To highlight the importance of thorough research into cannabinoid treatment, we aim to illustrate acute kidney injury (AKI). It is an example of a serious condition caused by synthetic cannabinoids. It is essential to emphasize the adverse impact of these substances on our body’s function. We have to keep in mind the importance of proper training and expertise in cannabinoid treatment.

AKI is described as a sudden loss of the kidneys’ ability to excrete. This condition can lead to irreversible loss of kidney cells and nephrons, which can potentially cause CKD. It can manifest as a mild and temporary or severe and persistent [160]. In AKI, acute tubular necrosis (ATN), resulting from ischemia or exposure to nephrotoxic substances, is a frequently encountered occurrence [161].

Ischemia and reperfusion (IR) injury is a major cause of AKI. It has been shown that kidney injury associated with reperfusion is correlated with increased levels of 2-AG in the kidneys, which may have significant clinical and therapeutic implications (Figure 2b) [114].

IR injury reduces endocannabinoid signaling and Na^+^/K^+^ ATPase expression and activity in LLC-PK1 cells (immortalized epithelial cell line derived from pig renal proximal tubules) and rat kidney cortexes. However, treatment with a CB1 or CB2 agonist reverses these effects in a CB1-dependent manner, indicating that CB1 activation may represent a novel pro-homeostatic mechanism, restoring proper Na^+^/K^+^ ATPase function following IR damage in renal proximal tubule cells [162].

New studies have demonstrated that AEA has renoprotective effects against mercuric chloride-induced kidney injury. It possesses anti-inflammatory, antioxidant, and anti-apoptotic properties, involving modulation of various molecular pathways including WNT-5A/BCL-2, IP3/NFATc1, HMGB-1/RAGE/NF-κB, and caspase cascades (Abdallah DM, Scientific Reports 2023) [163]. Furthermore, a study revealed that N-acylethanolamines such as AEA, N-oleoylethanolamine (OEA), and N-palmitoylethanolamine (PEA) may have a protective function against renal ischemia-induced injury [164]. These studies suggest potential therapeutic implications of the endocannabinoid system in addressing ischemia-related renal disorders.

One of the causes of AKI is synthetic cannabinoids, which are a type of psychoactive substance. They interact with the endocannabinoid system within many organs in the body. They also have the ability to react with CB1 and CB2. Activation of these receptors leads to the initiation of signaling pathways such as those for oxidative stress, inflammation, and apoptosis, which have the potential to cause detrimental changes in organs such as the liver, kidneys, heart, or brain. This can result in negative health consequences, including liver injury and damage, AKI, arrhythmias, myocardial infarction, and cognitive and motor impairment (Figure 2b) [165].

AKI associated with synthetic cannabinoids (SCs), such as K2, the brand name of one synthetic herbal mixture containing synthetic cannabinoids, often manifests as a sudden and severe condition. The majority of patients are young males who do not have any medical history of kidney disease. Typically, symptoms develop shortly after K2 intake. The most frequent manifestations of K2 ingestion are gastrointestinal problems such as nausea and vomiting. General constitutional symptoms occur as well. This condition, which ahs been increasingly observed in the United States, typically resolves rapidly with fluid resuscitation. SCs may accumulate in the kidneys due to the presence of CB1 and CB2 on podocytes in the human glomeruli [165].

The definitive pathophysiology of SC-associated AKI is still unspecified [166]. However, clinical evidence from renal biopsies and medical reports shows that ATN continues to be the most common pathology in AKI caused by K2 [165,167]. Specifically, SC XLR-11 has been identified as a nephrotoxic substance related to adverse effects on the mitochondrial function of proximal tubules [168].

A systematic review based on 55 young SC abusers who experienced AKI showed that renal damage was evidenced by elevated serum creatinine levels in 49 individuals, representing 89% of the cases [167]. Renal biopsy, conducted in 33% of the cases, unveiled acute tubular damage, acute tubulointerstitial nephritis, and acute interstitial nephritis, in descending order of occurrence. Nevertheless, diagnosing AKI related to SCs is a significant challenge in both the clinical and forensic fields. Considering these complexities, it is crucial to maintain a high level of suspicion for SC-related AKI, especially after ruling out other potential causes and when serum creatinine levels are elevated [167]. Due to SCs’ absence of standard blood and urine toxicology tests, nephrologists need to keep this in mind when diagnosing cases of unexplained AKI [168].

Additionally, a systematic review and meta-analysis conducted by Zihao Zhao found that targeting cannabinoid receptors can significantly improve kidney function in cases of renal dysfunction. Specifically, the blockade or knockout of CB1 significantly reduced blood urea nitrogen (SMD: −1.67), serum creatinine (SMD: −1.88), and albuminuria levels (SMD: −1.60). The activation of CB2 significantly reduced serum creatinine (SMD: −0.97) and albuminuria levels (SMD: −2.43) compared with the control group. These findings suggest that CB1 antagonists and CB2 agonists may reduce inflammatory responses and provide renal protective effects, maintaining therapeutic potential in various types of kidney disease [169].

In the United States, researchers analyzed data from 9,930,007 hospitalized patients with acute myocardial infarction (AMI) from the Nationwide Inpatient Sample from 1 January 2001 to 31 December 2020. Patients <18 and >80 years old were excluded. To differentiate the groups, cannabis users were compared with non-users. Those categorized as “drug abuse” based on ICD-9 and ICD-10 were excluded. Outcome differences were analyzed using multivariate regression models, which were adjusted for patient characteristics (e.g., sex, age, race, insurance, and weekend admission) and comorbidities such as hypertension, diabetes, smoking, dyslipidemia, chronic kidney disease, obesity, and prior cardiac interventions.

A total of 117,641 (1.2%) of these patients reported cannabis use. After conducting statistical tests and applying propensity score matching, they found that the cannabis users had a higher risk of developing acute kidney injury (aOR = 1.103, *p* < 0.01) [170].

The study was limited by the use of a modest CBG dose, early timing of assessments (20–60 min post dosing), and the remote Zoom-based methodology, which precluded physical exams, vital sign measurements, and open-ended adverse event reporting. Additionally, the sample consisted of experienced cannabis users rather than a clinical population, limiting generalizability, and multiple statistical comparisons were not corrected, increasing the potential for type I errors.

However, this study used a large, nationally representative sample spanning 20 years, which enhanced the generalizability and robustness of the findings. Additionally, it used rigorous statistical methods, including multivariate regression and propensity score matching, to adjust for confounding variables, ensuring more accurate comparisons between cannabis users and non-users, which makes this study valuable [170].

The abovementioned information highlights the critical need for recognizing the risks cannabinoids pose to kidney health. Ongoing research into cannabinoid receptor interactions offers promising therapeutic approaches, but careful attention and expertise are essential to safely manage these treatments.

## 5. Conclusions

This narrative review discussed the impact of various cannabinoids on kidney function. It covers new perspectives which are to be addressed in the future as well as the state of the art of molecular and animal model-based research.

Cannabis and its derivatives, including edibles and hemp seeds, are increasingly consumed for recreational, nutritional, and medicinal purposes, offering potential benefits but also posing risks such as inconsistent labeling, health implications, and overdosing. The growing prevalence of these products underscores the need for standardized regulations, accurate labeling, and further research to address health concerns, particularly for vulnerable conditions like kidney diseases.

Cannabinoid receptors CB1 and CB2 are present throughout the human body, including in the kidneys. More precisely, they are expressed in arterioles, proximal and distal tubules as well as podocytes and the mesangium, where their main role is controlling blood flow. Cannabinoids, both endogenous and exogenous, interact with CB1 and CB2 in the kidneys, affecting tubular function and renal parameters and thus acting similar to nutrients. Blocking CB1 has been shown to limit albuminuria, renal fibrosis, blood urea nitrogen, and serum creatinine, among other substances, which potentially could be useful in the treatment of various pathologies. These data seem even more promising with the acknowledgement of CB1 overexpression in DN and non-metabolic CKD. Activation of CB2 seems to act analogously to a CB1 blockade, which may explain its downregulation in kidney biopsies obtained from patients with advanced DM.

In contrast, the use of SCs is a major threat to renal health. Not only can they cause AKI, but their interaction with CBs can also have detrimental effects on other organs, as they were present in multiple cases among users. This underlines the importance of specifying what cannabinoid one is referring to when discussing the matter. Furthermore, there is a lack of evidence regarding both the long-term effects of these substances and the genetic factors’ role in the response to them in the context of kidney function.

Insufficient data regarding the subject is hampering repurposing of already-registered drugs and the submission for approval of others, as most of the presented effects have low grades of evidence. This state in turn impedes proper care of patients. Further studies should also focus on patients who choose to self-medicate with cannabinoids, as there remains a research gap regarding this group.

## Figures and Tables

**Table 1:** Names of selected cannabinoids and their effects on CB receptors.

Substance	Agonist or Antagonist	Source
**ENDOCANNABINOIDS**		
AEA	CB1/CB2 partial agonist	Justinová et al., Hanus et al. [56,60]
2-AG		
N-arachidonoyl dopamine	CB1/CB2 agonist	Bisogno et al. [61]
2-arachidonyl glyceryl ether	CB1 agonist	Hanus et al. [60]
Oleamide		Leggett et al. [62]
Virodhamine	CB1 antagonist (partial agonist in vitro) CB2 agonist	Fezza et al. [63]
**SYNTHETIC CANNABINOIDS**		
AKB48	CB1/CB2 agonist	Canazza et al. [64]
AM-251	CB1 antagonist	Barutta et al. [48]
AM-356	CB1/CB2 agonist	Nuria et al. [65]
AM-1220		Andersen et al. [66]
AM-694	CB1 agonist	Bertol et al. [67]
AM-2201	CB1/CB2 agonist	Corli et al. [68]
CP-47,497		Hruba et al. [69]
CP-55,940		Tomiyama et al. [70]
CRA-13		Gardin et al. [71]
Drinabant	CB1 antagonist	Zuurman et al. [72]
Hemopresine		Heimann et al. [73]
Ibipinabant		Lange et al. [74]
Hu-210	CB1/CB2 agonist	Martín-Calderón et. al. [75]
JWH-015	CB2 agonist	Fechtner et al. [76]
JWH-018	CB1/CB2 agonist	Tirri et al. [77]
JWH-081		Basavarajappa et al. [78]
JWH-122		Cannaert et al. [79]
JWH-250	CB1/CB2 agonist	Ossato et al. [80]
Otenabant	CB1 antagonist	Hadcock et al. [81]
RCS-4	CB1/CB2 agonist	Banister et al. [82]
RCS-8		Schubert et al. [83]
STS-135		Canazza et al. [84]
Rimonabant	CB1 antagonist	Huang et al. [85]
Surinabant		Rinaldi-Carmona et al. [86]
**PHYTOCANNABINOIDS**		
Cannabichromene (CBC)	CB/1CB2 partial agonist	Zagzoog et. al. [87]
Cannabidiol (CBD)	CB1/CB2 antagonist (negative allosteric modulator)	Laprairie et al. [24]
Cannabigerol (CBG)	CB1/CB2 partial agonist	Zagzoog et al. [87]
Cannabinol (CBN)		Rhee [88]
Tetrahydrocannabinol (THC)		Zagzoog et al. [87]

AEA = anandamide; CB = cannabinoid receptor; 2-AG = 2-arachidonoylglycerol; CBC = cannabichromene; CBD = cannabidiol; CBG = cannabigerol; CBN = cannabinol; THC = tetrahydrocannabinol.

## Data Availability

No new data were created or analyzed in this study. Data sharing is not applicable to this article.

## Abbreviations

2-AG	2-arachidonoylglycerol
AEA	Anandamide
AKI	Acute kidney injury
AMI	Acute myocardial infarction
ARA	Arachidonic acid
ATN	Acute tubular necrosis
CB	Cannabinoid receptor
CB1	Cannabinoid receptor 1
CB2	Cannabinoid receptor 2
CBC	Cannabichromene
CBD	Cannabidiol
CBE	Cannablesoin,
CBG	Cannabigerol
CBL	Cannabicyclol
CBM	Cannabinoid-based medication
CBN	Cannabinol
CBND	Cannabinodiol
CBT	Cannabitriol
CGRP	Calcitonin gene-related peptide
CKD	Chronic kidney disease
CNS	Central nervous system
COX	Cyclooxygenase
CUD	Cannabis use disorder
CYP	Cytochrome P450
DAG	DIacyloglycerol
DM	Diabetes mellitus
DN	Diabetic nephropathy
ERKs	Extracellular signal-regulated kinases
eGFR	Estimated glomerular filtration rate
FAAH	Fatty acid amide hydrolase
FXR	Farnesoid X receptor
GFR	Glomerular filtration rate
GPCR	G protein coupled receptor
IR	Ischemia and reperfusion
JNKs	C-Jun N-terminal kinases
LPS	Lipopolysaccharide
MAPK	Mitogen-activated protein kinase
NADA	N-arachidonoyl dopamine
NAPE	N-arachidonoyl phosphatidylethanolamine
PIP2	4,5-bisphosphate
PE	Phosphatidylethanolamine
PPAR	Peroxisome proliferator-activated receptor
PLC	Phospholipase C
SCs	Synthetic cannabinoids
THC	Delta-9-tetrahydrocannabinol
TLR	Toll-like receptor
TRPV1	Transient receptor potential cation channel vanilloid 1
Δ8-THC	Δ8 -trans-tetrahydrocannabinol
11-OH-THC	11-Hydroxy-Δ9-tetrahydrocannabinol
11-COOH-THC	11-Nor-9-carboxy-Δ9-tetrahydrocannabinol
7-OH-CBD	7-Hydroxy-CBD
